# Supplementary material for: An adaptive data-driven architecture for mental health care applications
Source: PeerJ. 2024 Mar 29;12:e17133. doi: 10.7717/peerj.17133 (PMC10984189; doi:10.7717/peerj.17133)
Supplement: Supplemental Information 2 [file peerj-12-17133-s002.pdf]

Systematic review and/or meta-analyses rationale and contribution

Index	Information requested by journal	Information provided by authors
1	The rationale for conducting the systematic review / meta-analysis;	The rationale behind this research is to create a versatile architecture for mental health care that harnesses the power of data-driven techniques and ensemble machine learning models. The aim is to efficiently structure, process, and exhibit data in the realm of positive computing. Through this adaptable data-driven architecture, it becomes possible to tailor interventions to address different categories of mental health disorders. The implementation of this decision-oriented machine learning approach has the potential to yield improved results in mental health care. It can also expand the accessibility of assistance for individuals managing a diverse range of mental health disorders, simultaneously aiding in the identification of intricate patterns that might be challenging for mental health experts to discern.
2	The contribution that it makes to knowledge in light of previously published related reports, including other meta-analyses and systematic reviews.	As mentioned in our introduction, there were limited studies concentrating on applications in mental health care. Based on our background study of existing literary works, most of the articles and systematic reviews primarily addressed patient health records, health referrals, disease diagnosis, or data analysis, rather than the combination of mental health disorders and data analytics. The contribution in "An adaptive data-driven architecture for mental health care applications" lies in its innovative approach of utilizing data-driven techniques to create a flexible framework for mental health care through the design of dedicated machine learning decision support layer. By translating the intricate patterns and characteristics of mental health instruments into machine-learning features, the application gains the ability to recognize and interpret important factors associated with mental well-being that might not be immediately apparent to mental health professionals. To the best of our knowledge, we have conducted a unique case study titled "Pandemic Anxiety Prediction Application." This approach represents a pioneering effort in proactively predicting and monitoring anxiety disorders during the onset of a pandemic, moving beyond the conventional focus solely on experimenting anxiety disorders.
